# Molecular Mechanisms of REM Sleep

**DOI:** 10.3389/fnins.2019.01402

**Published:** 2020-01-14

**Authors:** Rikuhiro G. Yamada, Hiroki R. Ueda

**Affiliations:** ^1^Laboratory for Synthetic Biology, RIKEN Center for Biosystems Dynamics Research, Osaka, Japan; ^2^Department of Systems Pharmacology, Graduate School of Medicine, The University of Tokyo, Tokyo, Japan

**Keywords:** REM sleep, theta oscillation, hippocampus, bursting, muscarinic acetylcholine receptors

## Abstract

Rapid-eye movement (REM) sleep is a paradoxical sleep state characterized by brain activity similar to wakefulness, rapid-eye-movement, and lack of muscle tone. REM sleep is a fundamental brain function, evolutionary conserved across species, including human, mouse, bird, and even reptiles. The physiological importance of REM sleep is highlighted by severe sleep disorders incurred by a failure in REM sleep regulation. Despite the intense interest in the mechanism of REM sleep regulation, the molecular machinery is largely left to be investigated. In models of REM sleep regulation, acetylcholine has been a pivotal component. However, even newly emerged techniques such as pharmacogenetics and optogenetics have not fully clarified the function of acetylcholine either at the cellular level or neural-circuit level. Recently, we discovered that the G_*q*_ type muscarinic acetylcholine receptor genes, *Chrm1* and *Chrm3*, are essential for REM sleep. In this review, we develop the perspective of current knowledge on REM sleep from a molecular viewpoint. This should be a starting point to clarify the molecular and cellular machinery underlying REM sleep regulation and will provide insights to explore physiological functions of REM sleep and its pathological roles in REM-sleep-related disorders such as depression, PTSD, and neurodegenerative diseases.

## Introduction

Rapid-eye movement (REM) sleep is a prominent brain state which is accompanied with multiple features such as random movements of eyes, vivid dreaming, quiet muscle tone, lessened homeostatic regulation of body (e.g., temperature, heart rate, etc.), and brain activity marked by the enhancement of specific brain oscillation. As indicated by the multiple features of REM sleep, the mechanisms of sleep, in general, involve multiple regulatory components at different layers of scales ranging from molecule level to organism level. The brain oscillation is an electrophysiological activity widely used to define stages of sleep. The identification of the brain oscillation associated with specific sleep stages has enabled researchers to untangle such intermingled systems in consideration of neural activity of the brain. There are largely three classes of oscillations in the brain of sleeping mice; slow-wave oscillation (SWO, <1 Hz), delta oscillation (1–4 Hz), and theta oscillation (4–10 or 12 Hz; this range slightly varies depending on literature). The SWO and delta oscillation are characteristic to non-REM (NREM) sleep, and the theta oscillation is characteristic to REM sleep. The early investigations into the SWO and delta oscillations provided the basis for current understanding of molecular and cellular mechanisms of NREM sleep. In contrast to the relatively well-described mechanisms of NREM sleep, that of REM sleep has been left unclear. Looking back some historical milestones of sleep research is helpful to grasp how the brain oscillations became established as definitive features of sleep stages and contributed to the current understanding of the mechanisms of NREM sleep. This retrospective detour into NREM sleep studies should be beneficial to make extrapolations on how the brain oscillations provide insights into the mechanisms of REM sleep.

The first measurement of the electrical activity of brain dates back to 1875 when the pioneer electrophysiologist; Caton (1875) reported that the electrical current in a cortical region varies depending on the functional activity of the region using dog, rabbit, and monkeys. Notably, he already described that the suspension of functional activity increased the electrical current, and the current diminished when the cortical area was functionally active (Cohen of Birkenhead, 1959). His notion is consistent with today’s knowledge that the sleeping cortex shows the relatively high amplitude of SWO and delta oscillation while the waking brain shows low amplitude fast oscillation. Without knowing Caton’s work, in 1890, Beck (1890) observed spontaneous rhythms of electrical brain activity and that the rhythm changes upon sensory stimulation using frogs, dogs, and rabbits. In 1910s, W. Práwdicz-Neminski identified the distinguishable patterns of the spontaneous rhythms and referred them as A-waves and B-waves using dogs (Coenen and Zayachkivska, 2013). In 1920s, the first electrical recording of a human brain was made by Berger (1929), who called his method “electro encephalogram (EEG).” He also observed distinct patterns of spontaneous rhythms which consisted of slow and fast oscillations. The slow and fast oscillations are around 10 and 30 Hz, respectively. Berger (1929) referred those oscillations as alpha and beta waves and reported that the alpha wave was replaced with the beta wave in response to physiological stimuli. Importantly, his extensive investigation of brain oscillations in various pathological and pharmacological conditions revealed that the slower alpha wave, which is referred as delta oscillation in today’s term, appeared when the subject was unconscious under anesthesia or epilepsy (Walter, 1938). The term of “delta wave” was first introduced by Walter in 1936 to describe slow oscillations produced in a cortical region adjacent to cerebral tumors when he studied the location of tumors by using EEG signal. Later, Loomis et al. (1937) used the term (delta wave) to describe the slow oscillations discovered in natural sleep of human and many other conditions (Walter, 1938). Loomis et al. (1937) built a large recording drum of EEG to observe a human subject continuously throughout a night, and they discovered several distinct stages of sleep and characteristic brain oscillations (Davis et al., 1937; Loomis et al., 1937). Later, these findings led to the identification of the sleep stage associated with REM and frequent dreaming (Aserinsky and Kleitman, 1953), and to the formulation of an objective method for studying sleep (Dement and Kleitman, 1957a, b). Since those seminal studies, EEG signal has been the definitive information for staging sleep. However, the understanding of the neuronal and molecular basis that underlie the characteristic EEG signals had to wait for the works of Steriade that attributed the high amplitude and low-frequency EEG oscillations, that is SWO and delta oscillation, to the synchronized activity of the population of neocortical and thalamocortical neurons (Steriade et al., 1993a,c,d).

Importantly, all of the three brain oscillations, SWO, delta oscillation, and theta oscillation, are originated from synchronized activity of a population of neurons. The cellular property shared among synchronous neurons is the bimodality of membrane potentials. The alternating sequence of the active state (UP state) and the silent state (DOWN state) rises to the brain oscillations. The UP state is associated with vigorous firings due to the depolarized membrane potential and the DOWN state associated with the ceasing of firing due to the hyperpolarized membrane potential (McCormick et al., 2015). Some early studies suggested that the essence of the alternating sequence is the interaction between the relatively slow Ca^2+^ oscillation and the fast action potentials, and suggested ion channels involved in the slow oscillation and action potentials (Jahnsen and Llinas, 1984; Steriade et al., 1993b; McCormick and Bal, 1997). Following the description of the SWO and the delta oscillation in the neocortex and the thalamus which underly the NREM sleep, the identification of involved brain regions and their brain-wide neural circuits have rapidly advanced, and we see further advancement in that direction thanks to the recent advent of innovative techniques such as opto- and pharmacogenetics (Weber and Dan, 2016; Saper and Fuller, 2017; Scammell et al., 2017).

While the neural circuits are relatively well described, the molecular and cellular properties essential to sleep regulation have been less investigated in the last decades. Moreover, the function of even basic neurotransmitters in regulating the cellular properties for sleep, especially the necessity of acetylcholine for REM sleep has been controversial for decades. Early studies implied the importance of acetylcholine for REM sleep by demonstrating that the injection of cholinergic agonists into the brainstem induced REM sleep-like state (Cordeau et al., 1963; George et al., 1964). Also, acetylcholine release was found to be abundant in the brainstem during REM sleep (Kodama et al., 1990; Leonard and Lydic, 1997). Therefore, a long-standing hypothetical model of the transition between NREM sleep and REM sleep incorporated acetylcholine as the key factor (Hobson et al., 1975; McCarley and Hobson, 1975; Sakai et al., 2001). However, the necessity of acetylcholine for REM sleep has been elusive, because lesioning of brain regions such as cholinergic neurons in the basal forebrain (BF), the laterodorsal tegmentum (LDT), and the pedunculopontine tegmentum (PPT) in the brainstem results in relatively minor effects (Lu et al., 2006; Blanco-Centurion et al., 2007). Hence, a proposed model for regulating REM sleep incorporates GABAergic and glutamatergic neurons as its core components (Luppi et al., 2013). Although recent opto- and pharmaco-genetic approaches consolidated the role of acetylcholine in sleep regulation at neural-circuit level, the necessity of acetylcholine in REM sleep regulation remained unclear (Shi et al., 2015; Xu et al., 2015; Chen et al., 2016; Zant et al., 2016). Despite the controversy over the importance of cholinergic regulation on REM sleep, multiple lines of *in vivo* pharmacological evidence consistently indicated muscarinic acetylcholine receptors are important for REM sleep regulation. Muscarinic receptor agonists and acetylcholinesterase inhibitors increase REM sleep and shorten the REM latency (the time-delay of REM start after the NREM start) (Sitaram et al., 1976; Hohagen et al., 1993; Lauriello et al., 1993; Riemann et al., 1994). On the other hand, muscarinic receptor antagonist decreased REM sleep and lengthened the REM latency (Gillin et al., 1991; Rauniar et al., 1998; Kim and Jeong, 1999). Nonetheless, genetic approaches to assess the contribution of muscarinic receptors to sleep-regulation have been limited (Goutagny et al., 2005), leaving the molecular component in the regulation of REM sleep unidentified.

To obtain deeper insights into the molecular mechanism of REM sleep, we need to address two issues: (1) identifying specific molecular components among the family members of acetylcholine receptors and (2) understanding the molecular function in regulating cellular properties of the identified receptors. A recent comprehensive reverse genetic study revealed that the G_*q*_ protein-coupled muscarinic acetylcholine receptors, Chrm1 and Chrm3, are essential for REM sleep, as REM sleep and its associated enrichment of EEG theta oscillation could be hardly detected in *Chrm1* and *Chrm3* double-knockout (DKO) mice during sleep (Niwa et al., 2018). Also, a series of our studies suggested that the Ca^2+^-hyperpolarization pathway plays an important role in regulating cellular properties for the synchronized activity for NREM sleep, i.e., for the SWO and the delta oscillation. Because the synchronized activity of population of neurons is also a mechanism that underlies the theta oscillation; a definitive feature of REM sleep, the investigation into the molecular mechanisms involved in the synchronized activity should be a future direction of REM sleep research. In this review, we intend to give a perspective on molecular mechanisms of REM sleep by focusing on the EEG theta oscillation in sleeping mice. First, we summarize the cellular basis of synchronized neurons underling the SWO and the delta oscillations, then we shift our viewpoint to the theta oscillation and look neural circuits involved in generation and regulation of the theta oscillation. We also discuss the current views about the function of *Chrm1* and *Chrm3* in the theta oscillation, and a potential molecular basis for sleep homeostasis. Previous excellent reviews have extensively described the regulatory neural circuits of REM sleep, the characteristic muscle activities associated with REM sleep such as REM and muscle atonia, the evolutional perspective of REM sleep, or the mechanism and function of SWO in the neural network (Brown et al., 2012; Luppi et al., 2013; Neske, 2015; Miyazaki et al., 2017). Here, we put our focus on the molecular mechanisms of EEG brain oscillations.

## The Cellular Mechanism of the Brain Oscillations

**FIGURE 1:**
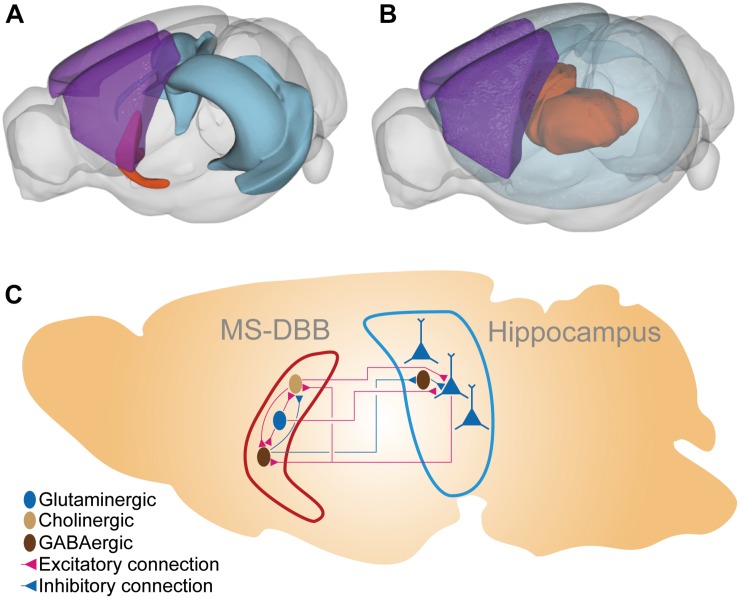
The brain region and the neural connections in the MS-DBB–hippocampus system. **(A)** The MS-DBB–hippocampus system depicted in 3D space. The blue region is the hippocampal formation; the orange region is the medial septal complex (including MS-DBB). The purple region is the somatomotor areas in the neocortex (isocortex) presented for visual aid to highlight the deep locations of the hippocampus and the medial septal complex. **(B)** The thalamocortical system depicted in 3D space. The transparent blue region represents the neocortex. The orange region represents thalamus. The purple region is the somatomotor areas in the neocortex presented for visual aid to highlight the deep location of the thalamus. **(C)** Schematic diagram of the MS-DBB–hippocampus system. The hippocampal pyramidal neurons (blue triangles) are aligned in parallel so that they produce the strong alteration in the electrical field measurable by EEG. The pyramidal neurons receive excitatory cholinergic and glutamatergic inputs from the MS-DBB and inhibitory inputs from hippocampal interneurons. The pyramidal neurons innervate cholinergic and GABAergic neurons in MS-DBB. Dark brown ellipsoids represent GABAergic neurons; light brown ellipsoids represent cholinergic neurons; blue ellipsoids and triangles represent glutaminergic neurons. Red connection and blue connections represent excitatory and inhibitory connections, respectively. The 3D plots were drawn with cocoframer available at a public mouse brain atlas for parts **(A)** and **(B)** (Allen Institute for Brain Science, 2018).

Although any cell types in the brain may contribute to the EEG signal as ionic flows are generated, the primary contributor is the pyramidal neuron in the neocortex. The neocortical pyramidal neurons reside under the skull, aligned in parallel to each other, and have thick dendrite that can form strong dipoles along the somatodendritic axis. The synchronized activation of those layered pyramidal neurons generates the strong extracellular electrical field readily measurable on the skull (Buzsaki et al., 2012). In contrast, the cerebellum which also has the layer structure of giant Purkinje neurons generate very small extracellular fields because the cerebellar activity is mainly local, and the Purkinje cells are not synchronized. Thalamocortical cells, which have more circular morphology emanating dendrites in all directions with relatively equal size compared to pyramidal neurons, can form limited dipoles and their contribution to extracellular fields is small even when their action is highly synchronized. Besides the neocortex, the hippocampus is a major influencer on the EEG signal. The hippocampus has a layered structure, hippocampal pyramidal neurons are densely aligned in parallel, and they act in a synchronized manner to generate strong electrical fields (Colgin, 2013, 2016). Because the hippocampus is located deeper in the brain compared to the neocortex, the detailed recording of the extracellular electrical field requires deep electrodes placed close to the tissue (Figures 1A,B). However, the characteristic oscillation at the theta frequency band (4–10 Hz) recorded in EEG is thought to originate from the hippocampus. Supporting evidence includes that the average magnitude of theta power measured by multisite recordings along the hippocampus–neocortex axis monotonically decreased with distance from the hippocampus and that the distribution of theta power on the neocortical surface reflects the physical layout of the underlying hippocampus (Bland and Whishaw, 1976; Sirota et al., 2008).

### Molecular Mechanisms of the Bimodality: UP and DOWN States of the Burst Firing

**FIGURE 2:**
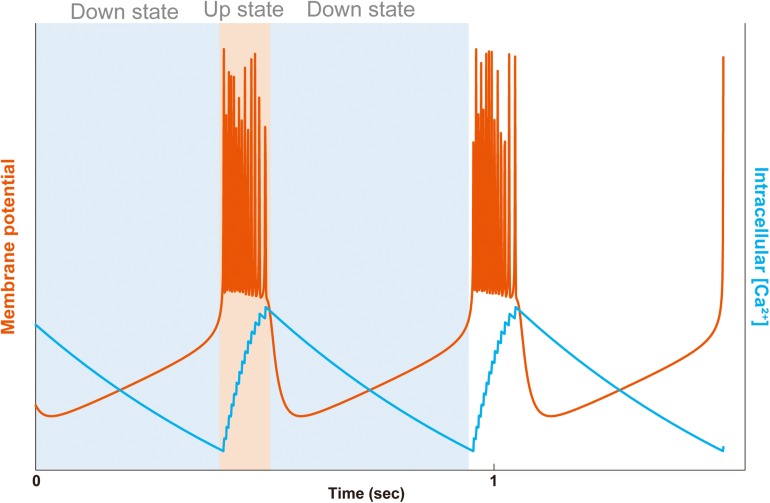
Bursting and intracellular Ca^2+^ concentration in a simple model. The membrane potential (orange line) and the intracellular Ca^2+^ concentration (blue line) are plotted. The bursting consists of UP and DOWN states alternating with each other. In the DOWN state (light blue background), the membrane potential is hyperpolarized to be quiet, and in the UP state (light orange background), the membrane potential is depolarized to generate a series of fast action potentials. In the first DOWN state, deactivation of the Ca^2+^-dependent potassium outward current (*I*_*K[Ca]*_) raise the membrane potential toward the threshold of Na^+^ and K^+^ dependent fast action potentials (*I*_*Na/K*_), entering the UP state. In the UP state, the fast Na^+^ spikes also activate high-threshold Ca^2+^ current (*I*_*Ca*_), building up the intracellular Ca^2+^ concentration. At the end of the UP state, due to the activation of the Ca^2+^-dependent potassium current (*I*_*K[Ca]*_), the membrane potential repolarizes, going to the DOWN state. In the second DOWN state, the hyperpolarized membrane then deactivates the *I*_*K[Ca]*_ to evoke another raise of membrane potential. The plot was calculated by the simplified-average neuron (SAN) model with arbitrary *y*-axis scales for visual aid (Yoshida et al., 2018).

The synchronized activity of neurons emerges from the bursting of individual neurons. The bursting consists of repeating cycles of the vigorous-firing state (UP state) and the silent state (DOWN state). The cycle can be observed in the neocortex and thalamus where a population of neurons synchronously generate the SWO and delta oscillation, respectively. The transition between UP and DOWN states is marked by a clear change of membrane potential, which traces the bimodality of neurons (Figure 2). The depolarized membrane potential underlies the UP state, and the hyperpolarized membrane potential underlies the DOWN state (Crunelli and Hughes, 2010). Some early studies focused on thalamic cells and depicted the molecular mechanism underlying the bimodality. In the classical explanation, the burst firing occurs from a hyperpolarized membrane potential. A burst firing consists of a series of ionic flows: (1) the hyperpolarization activated-cation channels (HCN) depolarizes the membrane (*I*_*h*_) to activate the transient slow low-threshold Ca^2+^ spike (*I*_*T*_), (2) the low-threshold Ca^2+^ spike triggers action potentials consisting of the fast in- and out-flow of sodium (*I*_*Na*_) and potassium (*I*_*K*_). In addition, the fast Na^+^ spikes also activate high-threshold Ca^2+^ current (*I*_*Ca*_), (3) after the burst of action potentials, the membrane repolarizes as the low-threshold Ca^2+^ spike (*I*_*T*_) ceases, (4) and the reduced depolarizing effect of *I*_*T*_ is followed by the overshooting after-hyperpolarization which is caused by the outflows of K^+^ due to the activation of Ca^2+^ dependent potassium channels (*I*_*K[Ca]*_) (Jahnsen and Llinas, 1984; McCormick and Bal, 1997). The essence of this ionic-flow model is the interaction between the relatively slow Ca^2+^ oscillation and the fast action potentials (Steriade et al., 1993b). Synaptic input is not critical part of the model. Indeed, the isolated thalamic neuron displays firing patterns similar to those of intact neurons in slice or *in vivo* (Hernandez-Cruz and Pape, 1989; Suzuki and Rogawski, 1989), and simple theoretical models generate bursting without explicit synaptic connections (Izhikevich, 2007).

In contrast to the established model of the cellular mechanism for the thalamic delta oscillation, cellular mechanisms for the neocortical SWO are less understood. This disparity is presumably because the neocortical SWO has been widely accepted to emerge from a finely tuned neural circuit consisting of balanced excitatory and inhibitory synaptic connections (Shu et al., 2003; Crunelli et al., 2015). One piece of supporting evidence of the view comes from the observation that the application of antagonist of non-NMDA glutamate receptor diminishes the SWO in the neocortical slices (Sanchez-Vives and McCormick, 2000). However, this view does not exclude the possibility that the ionic-flow mechanism similar to thalamic neurons also works in the neocortex. Indeed, the bursting persists in at least two groups of neocortical neurons even without synaptic connections at the frequency range of SWO (Le Bon-Jego and Yuste, 2007). Moreover, the neocortical bursting neurons have the electrophysiological properties characteristic to specific neurons that exhibit the slow Ca^2+^ oscillation (low-threshold Ca^2+^ spike), such as the rebound burst of action potentials following negative current injection (Huguenard, 1996; Le Bon-Jego and Yuste, 2007). Although it is to be confirmed that the intrinsic oscillatory property of neocortical cells is relevant to natural sleep, the property as ubiquitous as the low-threshold spike observed throughout brain regions with synchronized bursting for functions seems to play an important role in the neocortex (Huguenard, 1996; Li et al., 2009). Notably, it is recently shown that the changes in the composition of cortical interstitial Ca^2+^ and K^+^ ions influence the sleep–wake cycle (Ding et al., 2016). This study suggests that the intrinsic properties of neural oscillation may depend on the intracellular concentration of Ca^2+^ and K^+^ ions, which is in line with the observations that the loss of Ca^2+^ and K^+^ channels, such as *SK2* (*Kcnn2*) and *SK3* (*Kcnn3*), *Cav3.1* (*Cacna1g*), *Cav3.2* (*Cacna1h*), and *TASK3* (*Kcnk9*), affected the sleep duration *in vivo* (Tatsuki et al., 2016; Yoshida et al., 2018).

### Ca^2+^-Dependent Hyperpolarization Pathway for SWO

The investigations with emphasis on the circuit of SWO have provided the detailed view of intra-neocortical network of glutamatergic excitatory neurons and GABAergic inhibitory neurons and the inter-regional interaction between the thalamus and the neocortex (Crunelli et al., 2015; Neske, 2015). The circuit-based investigation has also been successful in describing the brain-wide regulatory neural circuits of sleep and wake cycle (Weber and Dan, 2016; Saper and Fuller, 2017; Scammell et al., 2017). On the other hand, the essential properties of oscillatory neurons remain elusive due to the complex nature of the interaction within the circuit. One approach is to simplify the neural circuit by constructing a computational model of “averaged” homogenous population of neurons (Tatsuki et al., 2016). The averaged-neuron (AN) model includes the excitatory glutamatergic AMPA and NMDA receptors that mediate Na^+^ and Ca^2+^ currents (*I*_*AMPA*_, *I*_*NMDA*_), respectively, and the inhibitory GABA_*A*_ receptors that regulate Cl^–^ currents (*I*_*GABA*_). The model also contains voltage-gated Ca^2+^ current (*I*_*Ca*_), voltage-gated and persistent sodium current (*I*_*Na*_, *I*_*NaP*_), several types of K^+^ currents including voltage-gated (*I*_*K*_), leak (*I*_*L*_), fast A-type (*I*_*A*_), inwardly rectifying (*I*_*AR*_), slowly inactivating (*I*_*KS*_), and Ca^2+^-dependent potassium currents (*I*_*K[Ca]*_). The unbiased search over the almost 20 million sets of parameters demonstrated that the AN model could generate bursting in a homogenous population of neurons. Moreover, the model predicted that the cellular Ca^2+^ plays a pivotal role in the alternation between UP and DOWN states.

To validate the prediction of the model, a series of knockout (KO) mice were generated. The KOs covered following genes: Ca^2+^ dependent K^+^ channels (eight genes) including *SK2* (*Kcnn2*) and *SK3* (*Kcnn3*), the voltage-gated Ca^2+^ channels (10 genes) including *Cav3.1* (*Cacna1g*) and *Cav3.2* (*Cacna1h*), the plasma membrane calcium pumps (four genes) including *Atb2b3* (*PMCA3*), and NMDA receptors (seven genes) including *Nr3a* (*GRIN3A*) (Sunagawa et al., 2016; Tatsuki et al., 2016). The results demonstrated that the changes of sleep duration observed in the mutant mice were consistent with the predictions. In addition, the acute or chronic pharmacological inhibition of NMDA receptors (possibly *Nr1/Nr2b*) in WT mice reduced the duration of sleep, suggesting the contribution of *Nr1* or *Nr2b*, the KO of which is embryonically lethal, in sleep regulation (Tatsuki et al., 2016). Building upon these results, they further tested the role of the major calcium-dependent protein kinase, *calcium-/calmodulin-dependent protein kinase type II* (*CaMKII*) in sleep regulation. Among the four different subunits of the *CaMKII* tested, *CaMK2a* KO and *CaMK2b* were found to be involved in the regulation of sleep/wake cycle (Tatsuki et al., 2016). Taken together, these results provided a hypothesis that the Ca^2+^-dependent hyperpolarization pathway plays an important role in regulating sleep duration through modulating the neural bimodality.

The AN model demonstrated that theoretical models could provide fundamental insight into the complex nature of bursting neurons. However, the AN model, which contains 13 components, is too complicated to interpret its detailed mathematical structure. Because it was important to elucidate how the transition between UP and DOWN states occurs, mathematical analyses, for example, to reveal the currents responsible for the transition were demanded. This point was addressed by constructing a simplified AN (SAN) model (Yoshida et al., 2018). Bifurcation and detailed mathematical analyses of the SAN model predicted that leak K^+^ channels play a role in generating bursting. Furthermore, the following comprehensive phenotype assays with 14 KO mice of leak K^+^ channels family identified that *potassium two pore domain channel subfamily K member 9* (*Kcnk9*) gene is involved in sleep regulation, validating the prediction of the mathematical analysis (Yoshida et al., 2018). It is interesting to note that this data of the involvement of constant K^+^ currents suggested that the conductance of leak K^+^ channels may alter the threshold for transition from UP to DOWN state mediated by the Ca^2+^-dependent K^+^ channels. Collectively, the Ca^2+^-dependent hyperpolarization pathway and leak K^+^ channels are involved in regulating SWO. The mathematical insight given by the potential role for constant currents such as leak K^+^ currents in affecting the bifurcation explains that the other constant synaptic currents mediated by, for example, AMPA receptors and GABA receptors can contribute to wakefulness and sleep.

## The Neural Circuits of the EEG Theta Oscillation

The extensive multisite measurements of local field potentials in rodents found the hippocampus as the cardinal source of the theta oscillation (Green and Arduini, 1954). The hippocampal theta oscillation was later to be found associated with REM sleep (Jouvet, 1969). Since then, the hippocampal theta oscillation in sleeping animal is recognized as a definitive feature of REM sleep. The EEG theta oscillation is a summation of multiple signals generated by spatially distributed oscillators in the hippocampal–entorhinal regions, and the oscillation amplitude and phase vary as a function of behavior (Sirota et al., 2008; Buzsaki et al., 2012). Notably, the isolated hippocampal neurons can exhibit oscillations at the theta frequency band *in vitro* when it is bathed in acetylcholine or kainate receptor agonist (Williams and Kauer, 1997; Garner et al., 2005; Manseau et al., 2005; Fuller et al., 2007). Moreover, the hippocampus neurons, such as CA3 pyramidal neurons, exhibit low-threshold Ca^2+^ spike and bursting (Llinas, 1988; Huguenard, 1996). Therefore, it is plausible to assume that the neurons of hippocampus possess an intrinsic ability to generate the theta oscillation. Interestingly, a recent study showed that a majority of hippocampal neurons are self-oscillatory, and the properties of oscillation, including frequency, are affected by environmental ions and cellular Ca^2+^ (Penn et al., 2016). This effect occurs without changes in synaptic connectivity or neural circuit, suggesting that the intrinsic neural properties directly affect circuit-level oscillation.

Together, the body of evidence suggests that the brain oscillations, including hippocampal theta oscillation, originate from intrinsic cellular properties. The intrinsic oscillation resonates and is amplified in neural circuits to operate physiological function implemented in each brain region. This view is consistent with the observed function of neural circuits. The intensive studies on neural circuits for regulating REM sleep have revealed multiple brain regions and extracellular neurotransmitters. The following sections briefly review the neural circuits focusing on the regulation of hippocampal theta oscillation.

### The Brain Regions Involved in the Regulation of the Hippocampal Theta Oscillation

Similar to the model of SWO or delta oscillation which consists of interacting intrinsic oscillators in the neocortex or the thalamus (Crunelli et al., 2015), the widely accepted model of hippocampal theta oscillation adopted the view of neural circuits as an oscillatory unit. In the model, the interaction between the medial septum (MS) and diagonal band of Broca (MS-DBB) and hippocampus mediates the generation of theta oscillation (Figure 1; Brown et al., 2012; Teles-Grilo Ruivo and Mellor, 2013). The loss of major afferent input from MS-DBB abolishes the theta oscillation in the hippocampal–entorhinal regions in urethane-anesthetized animals or awake animals indicating that the MS-DBB plays a critical role in hippocampal oscillation (Brazhnik and Fox, 1997; Yoder and Pang, 2005).

The MS-DBB is a region of the BF and composed of a heterogeneous population of neurons including GABAergic, cholinergic, and glutamatergic neurons. The large portion of the GABAergic neurons exhibits bursting activity at the theta frequency while the cholinergic neurons have a low firing rate not related to the theta oscillation (Simon et al., 2006). However, the targeted lesion of cholinergic neurons in MS-DBB by 192 IgG-saporin injections reduces the amplitude of the hippocampal theta oscillation indicating that the cholinergic projection also plays a role in the hippocampal theta (Lee et al., 1994; Yoder and Pang, 2005). The selective lesion of GABAergic neurons and potentially other non-cholinergic neurons by kainic acid reduces the hippocampal theta oscillation more than cholinergic lesion. The combined lesion of cholinergic and GABAergic neurons almost eliminates the hippocampal theta oscillation (Yoder and Pang, 2005). In addition, the isolated MS-DBB can exhibit oscillation at the theta frequency band *in vitro* when it is bathed in acetylcholine agonist (Konopacki et al., 1987a, b; Goutagny et al., 2009; Pignatelli et al., 2012). These observations supported the view that the MS-DBB, especially the GABAergic projection, provides the hippocampus with critical inputs to mediate the theta oscillation (Manseau et al., 2005; Teles-Grilo Ruivo and Mellor, 2013).

**FIGURE 3:**
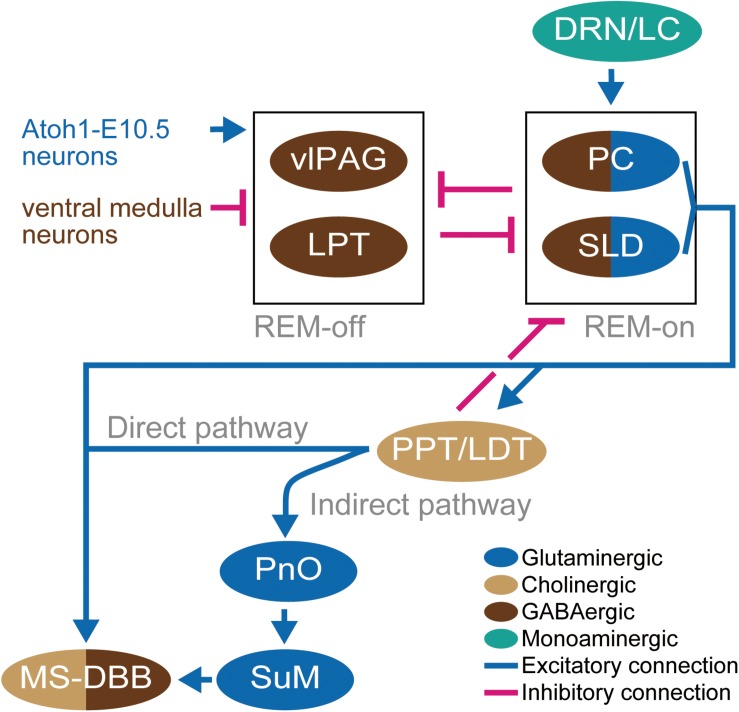
Schematic diagram of the neural circuit for EEG theta oscillation. Light brown ellipsoids represent cholinergic neurons, blue ellipsoids represent glutaminergic neurons, dark brown ellipsoids represent GABAergic neurons, and green ellipsoid represents monoaminergic neurons. Blue connections and red connections represent excitatory and inhibitory connections, respectively. Blue and dark brown letters for neurons represent glutamatergic and GABAergic neurons, respectively. The neurons represented by only letters are not depicted in ellipsoid because the definition of their residing brain regions has not been established yet.

The MS-DBB-hippocampus system has afferent input from nuclei in the brainstem from which the major sleep/wake regulatory pathways arise (Figure 3; Petsche et al., 1962; Buzsaki, 2002). The pons of the brainstem contains a population of REM-on neurons (i.e., neurons that are active during REM sleep) in the sublaterodosal nucleus (SLD), the REM-off neurons (i.e., neurons that are inactive during REM sleep) in ventrolateral periaqueductal gray matter (vlPAG), and the adjacent lateral pontine tegmentum (LPT) which is also known as the deep mesencephalic reticular nucleus (DpME) (Boissard et al., 2002; Lu et al., 2006; Scammell et al., 2017). The pathways ascend through the midbrain and then split into a dorsal pathway and ventral pathway. The dorsal pathway innervates the thalamus which projects to neocortical areas, while the ventral pathway innervates the BF including the MS-DBB, the hypothalamus, and the cortex (Brown et al., 2012).

### The Direct Projection From the Pons to MS-DBB

The cholinergic inputs from the brainstem are the major neuromodulators of MS-DBB (Mesulam et al., 1983). This cholinergic input consists of two different pathways (Figure 3). The one pathway is the direct cholinergic projection from the PPT and the LDT (Hallanger and Wainer, 1988). The activation of PPT and LDT by injection of glutamate results in neocortical desynchronization and hippocampal theta oscillation accompanied by wakefulness or REM sleep (Datta and Siwek, 1997). Additionally, the optogenetic activation of cholinergic neurons in PPT and LDT increased the initiation of REM sleep (Van Dort et al., 2015). However, selective lesions of PPT and LDT do not much affect REM sleep (Lu et al., 2006).

Importantly, retrograde tracer injected in the MS found positive cells in the precoerulueus (PC) region, the periventricular gray matter, and a dorsal extension of the SLD which shows high levels of activity (c-Fos expression) in REM sleep (Lu et al., 2006; Fuller et al., 2007). The projected PC neurons are glutamatergic, and the selective lesion of the PC and SLD abolished the theta oscillations during sleep (Lu et al., 2006), providing support for the concept that glutamatergic neurons in the PC regions play a critical role in mediating the emergence of theta oscillation from the cellular intrinsic oscillations in the MS-DBB–hippocampus system during REM sleep (Fuller et al., 2007). These observations give rise to the hypothesis of the “flip-flop model” in which the bidirectional inhibition between REM-on regions (PC and SLD) and REM-off regions (LPT and vlPAG) works like a flip-flop switch via GABAergic projections. The state of the switch is regulated by the excitatory inputs from serotoninergic dorsal raphe nucleus and locus coeruleus (DRN-LC) to the REM-on neurons (Lu et al., 2006), glutaminergic inputs from neurons located rostrolateral of SLD (Atoh1-E10.5 medial neurons) to REM-off neurons (Hayashi et al., 2015), and by the inhibitory inputs from the GABAergic ventral medulla neurons to REM-off neurons (Weber et al., 2015; Figure 3). The glutamatergic neurons in the PC and the dorsal part of the SLD project to the MS. On the other hand, the glutaminergic neurons in the ventral part of the SLD project to the spinal cord and regulate muscle atonia (Lu et al., 2006; Weng et al., 2014). Thus, lesions of the ventral SLD and PC produce a specific loss of REM sleep components; that is, the muscle atonia and the EEG theta, respectively (Fuller et al., 2007).

### The Indirect Projection From the Pons to MS-DBB

The other pathway is the indirect projection mediated by nuclei in the hypothalamus (Woodnorth et al., 2003; Figure 3). The supramammillary nucleus (SuM) in the hypothalamus is one candidate which may relay the regulation from the nucleus pontis oralis (PnO). PnO is a region of the brainstem reticular formation which is projected by PPT. The PnO activity is associated with the presence of hippocampal theta (Kirk and McNaughton, 1991; Oddie et al., 1994; Vertes and Kocsis, 1997; Pignatelli et al., 2012). Since the neurons in the PnO did not show rhythmic firing, the SuM has been assumed to translate the tonic firing of PnO into rhythmic firing. However, the lesion of SuM fails to affect theta rhythm (Thinschmidt et al., 1995; Renouard et al., 2015) while the inactivation of SuM by procaine injection reduces both frequency and amplitude of theta rhythm in the hippocampus (Kirk and McNaughton, 1993). The recent finding of the role for SuM in the creation of oscillatory interference between the theta oscillation of itself and the ongoing oscillations in its target areas suggested that the SuM is a coordinator of phase coherence of theta oscillations among brain regions (Ito et al., 2018).

## The Essential Genes for REM Sleep and the Associated EEG Theta Oscillation: *Chrm1* and *Chrm3*

**FIGURE 4:**
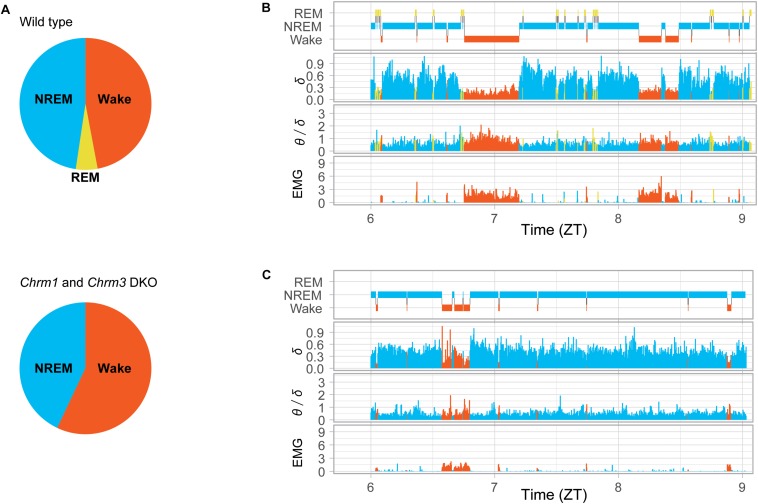
Power of EEG theta and delta oscillations in *Chrm1* and *Chrm3* DKO mice. **(A)** Pie charts presenting the proportions of sleep stages detected in wild-type mice (top) and double knockout (DKO) mice (bottom). REM sleep in wild-type mice was 72 min a day while almost undetected in DKO mice. **(B)** Hypnogram of a wild-type mouse. Delta power (normalized mV^2^), the ratio of theta/delta power, and total power of EMG signal. **(C)** Hypnogram of a *Chrm1* and *Chrm3* DKO mice. Orange for wakefulness, yellow for REM, and blue for NREM are shown. The enrichment of theta oscillation (increase in the value of θ/δ), that is associated with REM sleep in **(A)**, was hardly detected during sleep in the DKO mice **(C)**. The plots were reproduced from the data published in the literature (Niwa et al., 2018).

The accumulated evidence indicated that acetylcholine plays an important role in regulating REM sleep. However, it is demonstrated that the cholinergic function in a specific neural circuit can be limited (Grace et al., 2014). A possible function of acetylcholine is the regulation of cellular properties involved in the theta oscillation rather than of switching neural circuits. Indeed, isolated hippocampal and MS-DBB neurons can exhibit oscillations at the theta frequency band *in vitro* when it is bathed in acetylcholine receptor agonist (Williams and Kauer, 1997; Fellous and Sejnowski, 2000; Manseau et al., 2005). However, the molecular investigation into the necessity of acetylcholine for REM sleep has been hindered, because of the formidable redundancy resulting from the multitude of genes involved in the regulation: the 11 neuronal-type nicotinic acetylcholine receptors and 5 muscarinic acetylcholine receptors. The identification of critical cholinergic receptors has been unfeasible until the recent emergence of the efficient techniques in genetics such as CRISPR and ES-mice (Sunagawa et al., 2016; Ukai et al., 2017). The comprehensive study with these techniques on acetylcholine receptors revealed that DKO mice of G_*q*_ protein-coupled muscarinic acetylcholine receptors: *Chrm1* and *Chrm3* abolish REM sleep and the associated enrichment of EEG theta oscillation during sleep, leaving the theta oscillation largely unaffected during wakefulness (Niwa et al., 2018; Figure 4). This work demonstrated the necessity of acetylcholine for REM sleep and the EEG theta oscillation during sleep, which is in line with the previous pharmacological studies that demonstrated that the muscarinic blockers, such as atropine or scopolamine, diminish the EEG theta oscillation in anesthetized animals (Kim and Jeong, 1999; Buzsaki, 2002).

In contrast to the drastic sleep phenotypes observed in the DKO mice of muscarinic receptors, the comprehensive KO study of nicotinic acetylcholine receptors did not show significant sleep phenotype (Niwa et al., 2018). Nicotinic acetylcholine receptors are ionotropic, and their response is fast compared to the metabotropic muscarinic acetylcholine receptors. In consistent with the slow property of muscarinic acetylcholine receptors relevant to sleep regulation, the majority (80–90%) of cholinergic axon terminals in hippocampus are diffusely organized (Descarries et al., 1997), and do not associate with distinct postsynaptic sites suggesting that the cholinergic signaling in the hippocampus is primarily mediated by volume transmission as an ambient cholinergic tone instead of synaptic transmission (Teles-Grilo Ruivo and Mellor, 2013). Indeed, the elevated level of acetylcholine in the hippocampus is found associated with REM sleep *in vivo* (Teles-Grilo Ruivo et al., 2017). Together, these observations imply that acetylcholine contributes to the EEG theta oscillation of hippocampal neurons mainly through the slow regulation and modulate the intrinsic neuronal properties to generate the bursting activity at theta frequency band.

### The Possible Molecular Mechanism of the Theta Oscillation With *Chrm1* and *Chrm3*

The important insight given by the finding of the essential role for *Chrm1* and *Chrm3* in REM sleep is that, in contrast to the conventional neural-circuit view that the GABAergic input from MS-DBB neurons is primarily driving the EEG theta oscillation and the cholinergic regulation plays subsidiary role (Brown et al., 2012; Teles-Grilo Ruivo and Mellor, 2013), the cholinergic regulation in the MS-DBB–hippocampus system is comparably critical for the EEG theta oscillation at molecular and cellular level. The conventional view was based on the observations that the combined lesion of cholinergic and GABAergic neurons in MS-DBB almost eliminated the hippocampal theta oscillation (Yoder and Pang, 2005), theta activity survives in the hippocampus following the selective lesion of cholinergic neurons in MS-DBB (Lee et al., 1994), the selective lesion of GABAergic neurons and potentially other non-cholinergic neurons reduces the hippocampal theta oscillation more than cholinergic lesion (Yoder and Pang, 2005), and the large portion of the GABAergic neurons exhibits burst firing activity at the theta frequency while the cholinergic neurons have a low firing rate not related to the theta oscillation in MS-DBB (Simon et al., 2006). However, the almost complete absence of REM sleep in the *Chrm1* and *Chrm3 DKO* mice re-emphasized the role for the cholinergic pathway in REM sleep. Indeed, cultured hippocampal slice bathed in the acetylcholine agonist, carbachol, shows oscillation at the range of frequencies, including theta band depending on the drug concentration (Fellous and Sejnowski, 2000). The hippocampus intrinsic oscillation was inhibited either by *Chrm1* or *Chrm3* inhibitors (Williams and Kauer, 1997).

Although the current knowledge of the essential role for *Chrm1* and *Chrm*3 in REM sleep is based on the whole-body KO mice, investigations into the intrinsic cellular mechanisms involving *Chrm1* and *Chrm3* may contribute to understanding REM sleep regulation, much as the study of thalamocortical oscillations benefited from investigations into regulatory mechanisms of cellular excitability (Steriade et al., 1993b). In the thalamocortical cells, the membrane depolarization is mediated to a large part by the inhibition of a leak K^+^ conductance (*I*_*Kleak*_), the molecular instance of which is the two-pore domain K^+^ channels *TASK1* (*Kcnk3*) and *TASK3* (*Kcnk9*) (Meuth et al., 2006; Yoshida et al., 2018). The metabotropic glutamate and muscarinic acetylcholine receptors competitively activate G_*q*_ pathway, which in turn inhibit the leak K^+^ conductance (Chen et al., 2006; Coulon et al., 2010). A relatively small shift of membrane potential (∼10 mV) is sufficient to mediate a switch of firing mode *in vivo*. Moreover, the depolarization induced by the activation of muscarinic acetylcholine receptors *Chrm1* and *Chrm3* mediated by G_*q*_ proteins can mediate the switch in thalamocortical neurons (Broicher et al., 2008; Coulon et al., 2010). Because the level of acetylcholine is elevated in the hippocampus during REM sleep (Teles-Grilo Ruivo et al., 2017), it may be plausible to assume a similar mechanism works in the hippocampus to switch the hippocampal neurons between the oscillating modes. Notably, carbachol-induced depolarization of hippocampal CA1 neurons is eliminated from *Chrm1/3* DKO mice while the depolarization remained in single KO mice, suggesting that *Chrm1* and *Chrm3* receptors are each redundantly capable of depolarizing hippocampus neurons (Dasari and Gulledge, 2011). However, further studies are necessary to dissect the functions of *Chrm1* and *Chrm3* in the MS-DBB–hippocampus system.

### The Distinct Roles of *Chrm1* and *Chrm3* for REM Sleep

Comprehensive investigation on the acetylcholine receptors found that the combinatorial function of *Chrm1* and *Chrm3* is essential for REM sleep and associated EEG theta oscillation during sleep, whereas the function of each gene has yet to be investigated. Especially, single KO of either gene showed different phenotypic responses. The *Chrm1* KO mice had a reduced REM sleep duration, but NREM sleep duration was only moderately reduced. On the other hand, *Chrm3* KO mice had a reduced NREM sleep duration, but REM sleep duration was similar to that of WT mice (Niwa et al., 2018). These observations raised a question of what molecular mechanisms account for the observed difference. Muscarinic acetylcholine receptors consist of five isoforms and coupled with G_*q*_ proteins (M1, M3, and M5) or G_*i*_ proteins (M2 and M4). Differences in the preference of G protein coupling come from the difference in an amino acid sequence in the third intracellular loop between the M1, M3, and M5 sequences compared to the M2 and M4 sequences (Wess et al., 1997). However, several studies have shown that receptors coupling predominantly to one G protein family can also couple with other G proteins, though less efficiently. For example, Chrm3 receptor associates to both G_*q*_ and G_*i*_ in rat parotid glands (Dai et al., 1991), Chrm1 receptor also has G_*s*_ activity when ectopically expressed in Chinese hamster ovary (CHO) cells (Burford and Nahorski, 1996), whereas both Chrm1 and Chrm3 predominantly link to G_*q*_. In addition, a short variable sequence of the amphipathic helix (H8), typically three turns long and with palmitoylation sites at its C terminus, is present in several GPCRs including *Chrm3* (Qin et al., 2011; Venkatakrishnan et al., 2013). The H8 forms the preassembly with G_*q*_ proteins, which may contribute to the possible difference in the rate of receptor activation, compared with *Chrm1* (Qin et al., 2011). Another possibility is the different spatial distribution of *Chrm1* and *Chrm3* in the brain (Levey et al., 1995). Indeed, about 60% of the total muscarinic acetylcholine receptors of the hippocampus is Chrm1 receptors, whereas Chrm3 receptor is up to 10% (Dasari and Gulledge, 2011). Future investigation of downstream pathways from the identified receptors: *Chrm1* and *Chrm3* may reveal the mechanism of REM sleep and their physiological roles. Especially, several functions related to the long-term potentiation (LTP) have been shown to be mediated particularly by *Chrm1*. For example, *Chrm1*-dependent inhibition of SK channels enhances NMDA receptor function to facilitate the induction of LTP (Buchanan et al., 2010), and the *Chrm1*-dependent inhibition of voltage-activated Kv7 potassium channels facilitate LTP (Petrovic et al., 2012; Teles-Grilo Ruivo and Mellor, 2013), while involvement of *Chrm3* in this context is unknown.

## Phosphorylation Hypothesis for the Homeostatic Regulation of Sleep

The amount of sleep is regulated to be in a physiologically feasible range. This regulation is called the homeostatic regulation of sleep. The homeostatic regulation can comprise two distinct hypothetical processes: “process C” and “process S” (Borbely, 1982). Process C is the circadian component that regulates the propensity of sleep with the rhythm of 24 h. The process S is a sleep-dependent process that monitors accumulated amount of sleep and compensates the detected loss or excess of sleep. The mechanism of the homeostasis is under vigorous investigation at present.

### Homeostatic Regulation of NREM Sleep

The EEG power in the delta frequency band reflects the pressure for the NREM-sleep resulting from the loss of NREM sleep. The NREM-sleep need increases during wakefulness period, while it decreases during the sleep period. The changes of NREM-sleep need are well described by exponential function (Borbely, 1982). An apparent but unresolved question is by what molecular mechanism NREM-sleep need is represented. One important criterion to be satisfied is that the molecular mechanism must work in the time scale of minutes to hours, which is slower in order of magnitude compared to the time scale of neural action potentials. Candidate components of the mechanism include ion concentration, gene expression, post-translational modification, and production/degradation of ion channels or pumps.

Interestingly, in the context of Process C, the phosphorylation plays an important role in keeping the circadian period about 24 h (Tomita et al., 2005; Isojima et al., 2009; Shinohara et al., 2017). Phosphorylation was indicated to play a role also in Process S, as the loss of calcium/CaMKII gene resulted in significant reduction of sleep (Tatsuki et al., 2016). This finding is further supported by the observation that the wakefulness induced phosphorylation in the extracellular signal-regulated kinase (ERK) proteins, which are upstream of a group of genes expressed in activity-dependent manner and involved in sleep regulation (Mikhail et al., 2017). Moreover, the following phosphoproteomics studies revealed a number of genes in the intracellular signaling pathways change their states of phosphorylation along with the sleep/wake cycles (Diering et al., 2017; Wang et al., 2018; Bruning et al., 2019). Because protein functions can be modulated by site-specific phosphorylation or by cumulative phosphorylation of multiple sites, all of this evidence strongly suggests that the phosphorylation process is a component of the homeostatic regulation of sleep.

Because the Ca^2+^-dependent hyperpolarization pathway plays an important role in switching UP and DOWN states of neurons (Tatsuki et al., 2016, 2017), and the persistent UP state is associated with wakefulness, Ca^2+^-dependent phosphorylation is a promising regulatory component for the homeostasis in sleep and wake cycle (Ode et al., 2017; Shi and Ueda, 2018). A candidate gene family to be involved in Ca^2+^-dependent phosphorylation is calcium/CaMKII. Indeed, the KO mice of the CaMKII family revealed that either KO of CaMKIIα and CaMKIIβ results in significant reduction of sleep duration (Tatsuki et al., 2016), implying that CaMKII may be link between the actions of Ca^2+^ in the time domain of second to the activity of the kinase in the time domain of hours.

### Homeostatic Regulation of REM Sleep

In contrast to the EEG delta power established as an indicator of NREM-sleep need, any single component of EEG spectral power has not been established to represent REM-sleep need. Nonetheless, REM sleep is also under the homeostatic regulation; that is, the loss of REM sleep is compensated for by the increase in the duration of REM sleep (Franken, 2002). Selective REM sleep deprivation induces a rebound increase in subsequent REM sleep. Because the selective REM sleep deprivation does not largely affect the amount of NREM sleep, the homeostatic regulation of REM sleep seems to be likely independent to that of NREM sleep (McCarthy et al., 2016). However, prolonged REM sleep increases the delta power in the subsequent NREM sleep indicating there is profound interaction between REM and NREM sleep homeostatic regulatory mechanisms (Hayashi et al., 2015). Although the molecular machinery of the REM sleep homeostasis has yet to be investigated, it is plausible to assume mechanisms similar to the NREM sleep homeostasis, such as phosphorylation process, also work in REM sleep homeostasis. Notably, while most antidepressants suppress REM sleep, the physiologically induced REM sleep deficits are compensated for regardless of the subsequent pharmacological suppression of REM sleep (McCarthy et al., 2016). This observation implies that the homeostatic regulation of REM sleep consists of molecular and cellular mechanisms distinct from the neural circuits mediated by neurotransmitters, which are targeted by the antidepressants, such as serotonin and acetylcholine pathways. This insight is in line with the hypothetical involvement of the phosphorylation process in the homeostatic regulation, suggesting that the homeostasis is implemented at the cellular level rather than at the neural circuits level.

## Future Perspective

The pioneering studies focused on the electrophysiological activity of brains and identified characteristic EEG signatures such as SWO, delta, and theta oscillation to define sleep stages. The investigations revealed the underlying cellular level machinery to generate bursting activity for explaining the population level EEG signals. Based on those findings, in the last couple of decades, the focused study on the neural circuits of sleep regulation has been successful in identifying brain regions and detailed interactions among the regions (Scammell et al., 2017). However, the conventional way to investigate the electrophysiological properties of neurons has been mostly pharmacological approaches, hence an identified molecular component in the machinery is a cluster of molecules responsive to the applied drug. This restriction has hindered the identification of specific genes involved in sleep regulation. The recent advent of so-called next-generation genetics such as CRISPR and ES-mouse methods has been easing the long-standing restriction by significantly reducing the time and cost to produce KO or knockin mice of specifically targeted genes (Sunagawa et al., 2016; Ukai et al., 2017). Using these methods, researchers can generate a variety of KO mice covering the cluster of genes involved in a sub-system of sleep regulation. The application of the methods revealed that the genes involved in the Ca^2+^-dependent hyperpolarization pathway are important in sleep regulation (Sunagawa et al., 2016; Tatsuki et al., 2016; Yoshida et al., 2018). Besides identifying genes involved in NREM sleep duration, the next-generation genetics also identified genes essential for REM sleep: muscarinic acetylcholine receptors *Chrm1* and *Chrm3* (Niwa et al., 2018).

It is also notable that the reverse genetics approach demonstrated the role of the major calcium-dependent protein kinase, *CaMKII* in sleep regulation (Tatsuki et al., 2016), suggesting that the phosphorylation is involved in the sleep regulation. In addition to the reverse-genetic approach, the effort of forward-genetics also demonstrated that the mutation of Sik3 protein kinase gene causes a profound increase in sleep duration by a gain-of-function mutation (Funato et al., 2016). The phosphorylation process may occur in the time scale of hours, and be modulated by cumulative phosphorylation of multiple sites, e.g., casein kinase I (CKI) in the circadian regulation (Isojima et al., 2009; Shinohara et al., 2017). We note that the genes involved both in phosphorylation and sleep regulation are attractive candidates for the future studies on the homeostatic regulation of sleep (Ode et al., 2017; Tatsuki et al., 2017; Shi and Ueda, 2018).

The finding of the almost abolished REM sleep in the *Chrm1* and *Chrm3* DKO mice may provide a useful tool to clarify the function of REM sleep in learning and memory (Niwa et al., 2018). The dominance of theta oscillation in EEG signal during REM sleep indicates the synchronized activity of hippocampal neurons. The synchronization is believed to be critical for transferring information between neocortex and hippocampus and the sleep-related neural plasticity (Sirota et al., 2008; Grosmark et al., 2012). The optogenetic silencing of GABAergic neurons during REM sleep in the MS-DBB hindered the mice from properly consolidating what they learned prior to the sleep (Boyce et al., 2016), indicating the important roles for the theta oscillation in REM sleep and associated learning and memory. This function of theta oscillation is coinciding with that of delta or SWO in the thalamocortical system. SWO promotes learning and memory consolidation (Marshall et al., 2006; Miyamoto et al., 2016). On the other hand, a pharmacogenetic study revealed that reduction or induction of REM sleep attenuates or enhances SWO, respectively, in the subsequent NREM sleep (Hayashi et al., 2015). Thus, REM sleep might indirectly regulate memory formation in the neocortex through NREM sleep (Miyazaki et al., 2017).

Aside learning and memory in the hippocampus, other physiological functions of REM sleep remain obscure. Interestingly, the duration of REM sleep increases in some depression and the most antidepressants inhibit REM sleep in animals and humans (McCarthy et al., 2016). This strong correlation between REM sleep and psychiatric disorders including post-traumatic stress disorder (PTSD) implies that controlling REM sleep may help PTSD patients to alleviate the symptoms. The further elucidation of the molecular mechanism of theta oscillation will provide significant insights on how to control the amount of REM sleep both in mice and humans and may facilitate, for example, to refine antidepressants and to reveal the physiological roles of REM sleep in its closely related higher cognitive functions such as dreaming or consciousness.

## Author Contributions

All authors listed have made a substantial, direct and intellectual contribution to the work, and approved it for publication.

## Conflict of Interest

The authors declare that the research was conducted in the absence of any commercial or financial relationships that could be construed as a potential conflict of interest.

## References

[B1] Allen Institute for Brain Science (2018). *Allen Brain Explorer.* Available at: http://connectivity.brain-map.org/3d-viewer/ (accessed December 18, 2019).

[B2] Aserinsky E. Kleitman N. (1953). Regularly occurring periods of eye motility, and concomitant phenomena, during sleep. *Science* 118 273–274. 10.1126/science.118.3062.273 13089671

[B3] Beck A. (1890). The determination of the localisation of the brain and spinal cord functions by way of electrical appearances. *Cent. Physiol.* 4 473–476.

[B4] Berger H. (1929). Electroencephalogram in humans. *Arch. Fur Psychiat. Nervenkr.* 87 527–570. 10.1007/Bf01797193

[B5] Blanco-Centurion C. Gerashchenko D. Shiromani P. J. (2007). Effects of saporin-induced lesions of three arousal populations on daily levels of sleep and wake. *J. Neurosci.* 27 14041–14048. 10.1523/JNEUROSCI.3217-07.2007 18094243 PMC2975593

[B6] Bland B. H. Whishaw I. Q. (1976). Generators and topography of hippocampal theta (RSA) in the anaesthetized and freely moving rat. *Brain Res.* 118 259–280. 10.1016/0006-8993(76)90711-3 1000290

[B7] Boissard R. Gervasoni D. Schmidt M. H. Barbagli B. Fort P. Luppi P. H. (2002). The rat ponto-medullary network responsible for paradoxical sleep onset and maintenance: a combined microinjection and functional neuroanatomical study. *Eur. J. Neurosci.* 16 1959–1973. 10.1046/j.1460-9568.2002.02257.x 12453060

[B8] Borbely A. A. (1982). A two process model of sleep regulation. *Hum. Neurobiol.* 1 195–204. 7185792

[B9] Boyce R. Glasgow S. D. Williams S. Adamantidis A. (2016). Causal evidence for the role of REM sleep theta rhythm in contextual memory consolidation. *Science* 352 812–816. 10.1126/science.aad5252 27174984

[B10] Brazhnik E. S. Fox S. E. (1997). Intracellular recordings from medial septal neurons during hippocampal theta rhythm. *Exp. Brain Res.* 114 442–453. 10.1007/pl00005653 9187280

[B11] Broicher T. Wettschureck N. Munsch T. Coulon P. Meuth S. G. Kanyshkova T. (2008). Muscarinic ACh receptor-mediated control of thalamic activity via G(q)/G (11)-family G-proteins. *Pflugers Arch.* 456 1049–1060. 10.1007/s00424-008-0473-x 18350314

[B12] Brown R. E. Basheer R. McKenna J. T. Strecker R. E. McCarley R. W. (2012). Control of sleep and wakefulness. *Physiol. Rev.* 92 1087–1187. 10.1152/physrev.00032.2011 22811426 PMC3621793

[B13] Bruning F. Noya S. B. Bange T. Koutsouli S. Rudolph J. D. Tyagarajan S. K. (2019). Sleep-wake cycles drive daily dynamics of synaptic phosphorylation. *Science* 366:eaav3617. 10.1126/science.aav3617 31601740

[B14] Buchanan K. A. Petrovic M. M. Chamberlain S. E. Marrion N. V. Mellor J. R. (2010). Facilitation of long-term potentiation by muscarinic M(1) receptors is mediated by inhibition of SK channels. *Neuron* 68 948–963. 10.1016/j.neuron.2010.11.018 21145007 PMC3003154

[B15] Burford N. T. Nahorski S. R. (1996). Muscarinic m1 receptor-stimulated adenylate cyclase activity in Chinese hamster ovary cells is mediated by Gs alpha and is not a consequence of phosphoinositidase C activation. *Biochem. J.* 315(Pt 3), 883–888. 10.1042/bj3150883 8645172 PMC1217289

[B16] Buzsaki G. (2002). Theta oscillations in the hippocampus. *Neuron* 33 325–340. 11832222 10.1016/s0896-6273(02)00586-x

[B17] Buzsaki G. Anastassiou C. A. Koch C. (2012). The origin of extracellular fields and currents–EEG, ECoG, LFP and spikes. *Nat. Rev. Neurosci.* 13 407–420. 10.1038/nrn3241 22595786 PMC4907333

[B18] Caton R. (1875). The electric currents of the brain. *Br. Med. J.* 2 278.

[B19] Chen L. Yin D. Wang T. X. Guo W. Dong H. Xu Q. (2016). Basal forebrain cholinergic neurons primarily contribute to inhibition of electroencephalogram delta activity, rather than inducing behavioral wakefulness in mice. *Neuropsychopharmacology* 41 2133–2146. 10.1038/npp.2016.13 26797244 PMC4908644

[B20] Chen X. Talley E. M. Patel N. Gomis A. McIntire W. E. Dong B. (2006). Inhibition of a background potassium channel by Gq protein alpha-subunits. *Proc. Natl. Acad. Sci. U.S.A.* 103 3422–3427. 10.1073/pnas.0507710103 16492788 PMC1413874

[B21] Coenen A. Zayachkivska O. (2013). Adolf beck: a pioneer in electroencephalography in between Richard Caton and Hans Berger. *Adv. Cogn. Psychol.* 9 216–221. 10.2478/v10053-008-0148-3 24605179 PMC3902832

[B22] Cohen of Birkenhead (1959). Richard Caton (1842-1926) pioneer electrophysiologist. *Proc. R. Soc. Med.* 52 645–651. 10.1177/003591575905200816 19994019 PMC1870055

[B23] Colgin L. L. (2013). Mechanisms and functions of theta rhythms. *Annu. Rev. Neurosci.* 36 295–312. 10.1146/annurev-neuro-062012-170330 23724998

[B24] Colgin L. L. (2016). Rhythms of the hippocampal network. *Nat. Rev. Neurosci.* 17 239–249. 10.1038/nrn.2016.21 26961163 PMC4890574

[B25] Cordeau J. P. Moreau A. Beaulnes A. Laurin C. (1963). Eeg and behavioral changes following microinjections of acetylcholine and adrenaline in the brain stem of cats. *Arch. Ital. Biol.* 101 30–47. 14135066

[B26] Coulon P. Kanyshkova T. Broicher T. Munsch T. Wettschureck N. Seidenbecher T. (2010). Activity modes in thalamocortical relay neurons are modulated by G(q)/G(11) family G-proteins - serotonergic and glutamatergic signaling. *Front. Cell Neurosci.* 4:132. 10.3389/fncel.2010.00132 21267426 PMC3024565

[B27] Crunelli V. David F. Lorincz M. L. Hughes S. W. (2015). The thalamocortical network as a single slow wave-generating unit. *Curr. Opin. Neurobiol.* 31 72–80. 10.1016/j.conb.2014.09.001 25233254

[B28] Crunelli V. Hughes S. W. (2010). The slow (1 Hz) rhythm of non-REM sleep: a dialogue between three cardinal oscillators. *Nat. Neurosci.* 13 9–17. 10.1038/nn.2445 19966841 PMC2980822

[B29] Dai Y. S. Ambudkar I. S. Horn V. J. Yeh C. K. Kousvelari E. E. Wall S. J. (1991). Evidence that M3 muscarinic receptors in rat parotid gland couple to two second messenger systems. *Am. J. Physiol.* 261(6 Pt 1), C1063–C1073. 10.1152/ajpcell.1991.261.6.C1063 1722644

[B30] Dasari S. Gulledge A. T. (2011). M1 and M4 receptors modulate hippocampal pyramidal neurons. *J. Neurophysiol.* 105 779–792. 10.1152/jn.00686.2010 21160001 PMC3059175

[B31] Datta S. Siwek D. F. (1997). Excitation of the brain stem pedunculopontine tegmentum cholinergic cells induces wakefulness and REM sleep. *J. Neurophysiol.* 77 2975–2988. 10.1152/jn.1997.77.6.2975 9212250

[B32] Davis H. Davis P. A. Loomis A. L. Harvey E. N. Hobart G. (1937). Changes in human brain potentials during the onset of sleep. *Science* 86 448–450. 10.1126/science.86.2237.448 17838964

[B33] Dement W. Kleitman N. (1957a). Cyclic variations in EEG during sleep and their relation to eye movements, body motility, and dreaming. *Electroencephalogr. Clin. Neurophysiol.* 9 673–690. 10.1016/0013-4694(57)90088-3 13480240

[B34] Dement W. Kleitman N. (1957b). The relation of eye movements during sleep to dream activity: an objective method for the study of dreaming. *J. Exp. Psychol.* 53 339–346. 10.1037/h0048189 13428941

[B35] Descarries L. Gisiger V. Steriade M. (1997). Diffuse transmission by acetylcholine in the CNS. *Prog. Neurobiol.* 53 603–625. 10.1016/s0301-0082(97)00050-6 9421837

[B36] Diering G. H. Nirujogi R. S. Roth R. H. Worley P. F. Pandey A. Huganir R. L. (2017). Homer1a drives homeostatic scaling-down of excitatory synapses during sleep. *Science* 355 511–515. 10.1126/science.aai8355 28154077 PMC5382711

[B37] Ding F. O’Donnell J. Xu Q. Kang N. Goldman N. Nedergaard M. (2016). Changes in the composition of brain interstitial ions control the sleep-wake cycle. *Science* 352 550–555. 10.1126/science.aad4821 27126038 PMC5441687

[B38] Fellous J. M. Sejnowski T. J. (2000). Cholinergic induction of oscillations in the hippocampal slice in the slow (0.5-2 Hz), theta (5-12 Hz), and gamma (35-70 Hz) bands. *Hippocampus* 10 187–197. 10.1002/(sici)1098-1063(2000)10:2<187::aid-hipo8>3.0.co;2-m 10791841

[B39] Franken P. (2002). Long-term vs. short-term processes regulating REM sleep. *J. Sleep Res.* 11 17–28. 10.1046/j.1365-2869.2002.00275.x 11869422

[B40] Fuller P. M. Saper C. B. Lu J. (2007). The pontine REM switch: past and present. *J. Physiol.* 584(Pt 3), 735–741. 10.1113/jphysiol.2007.140160 17884926 PMC2276987

[B41] Funato H. Miyoshi C. Fujiyama T. Kanda T. Sato M. Wang Z. (2016). Forward-genetics analysis of sleep in randomly mutagenized mice. *Nature* 539 378–383. 10.1038/nature20142 27806374 PMC6076225

[B42] Garner H. L. Whittington M. A. Henderson Z. (2005). Induction by kainate of theta frequency rhythmic activity in the rat medial septum-diagonal band complex in vitro. *J. Physiol.* 564(Pt 1), 83–102. 10.1113/jphysiol.2004.080622 15677688 PMC1456035

[B43] George R. Haslett W. L. Jenden D. J. (1964). A Cholinergic mechanism in the brainstem reticular formation: induction of paradoxical sleep. *Int. J. Neuropharmacol.* 3 541–552. 14344492 10.1016/0028-3908(64)90076-0

[B44] Gillin J. C. Sutton L. Ruiz C. Golshan S. Hirsch S. Warmann C. (1991). Dose dependent inhibition of REM sleep in normal volunteers by biperiden, a muscarinic antagonist. *Biol. Psychiatry* 30 151–156. 10.1016/0006-3223(91)90169-m 1912107

[B45] Goutagny R. Comte J. C. Salvert D. Gomeza J. Yamada M. Wess J. (2005). Paradoxical sleep in mice lacking M3 and M2/M4 muscarinic receptors. *Neuropsychobiology* 52 140–146. 10.1159/000087560 16110248

[B46] Goutagny R. Jackson J. Williams S. (2009). Self-generated theta oscillations in the hippocampus. *Nat. Neurosci.* 12 1491–1493. 10.1038/nn.2440 19881503

[B47] Grace K. P. Vanstone L. E. Horner R. L. (2014). Endogenous cholinergic input to the pontine REM sleep generator is not required for REM sleep to occur. *J. Neurosci.* 34 14198–14209. 10.1523/JNEUROSCI.0274-14.2014 25339734 PMC6608391

[B48] Green J. D. Arduini A. A. (1954). Hippocampal electrical activity in arousal. *J. Neurophysiol.* 17 533–557. 10.1152/jn.1954.17.6.533 13212425

[B49] Grosmark A. D. Mizuseki K. Pastalkova E. Diba K. Buzsaki G. (2012). REM sleep reorganizes hippocampal excitability. *Neuron* 75 1001–1007. 10.1016/j.neuron.2012.08.015 22998869 PMC3608095

[B50] Hallanger A. E. Wainer B. H. (1988). Ascending projections from the pedunculopontine tegmental nucleus and the adjacent mesopontine tegmentum in the rat. *J. Comp. Neurol.* 274 483–515. 10.1002/cne.902740403 2464621

[B51] Hayashi Y. Kashiwagi M. Yasuda K. Ando R. Kanuka M. Sakai K. (2015). Cells of a common developmental origin regulate REM/non-REM sleep and wakefulness in mice. *Science* 350 957–961. 10.1126/science.aad1023 26494173

[B52] Hernandez-Cruz A. Pape H. C. (1989). Identification of two calcium currents in acutely dissociated neurons from the rat lateral geniculate nucleus. *J. Neurophysiol.* 61 1270–1283. 10.1152/jn.1989.61.6.1270 2501459

[B53] Hobson J. A. McCarley R. W. Wyzinski P. W. (1975). Sleep cycle oscillation: reciprocal discharge by two brainstem neuronal groups. *Science* 189 55–58. 10.1126/science.1094539 1094539

[B54] Hohagen F. Riemann D. Spiegel R. Holzhauer M. Berger M. (1993). Influence of the cholinergic agonist SDZ 210-086 on sleep in healthy subjects. *Neuropsychopharmacology* 9 225–232. 10.1038/npp.1993.58 8280346

[B55] Huguenard J. R. (1996). Low-threshold calcium currents in central nervous system neurons. *Annu. Rev Physiol.* 58 329–348. 10.1146/annurev.ph.58.030196.001553 8815798

[B56] Isojima Y. Nakajima M. Ukai H. Fujishima H. Yamada R. G. Masumoto K. H. (2009). CKIepsilon/delta-dependent phosphorylation is a temperature-insensitive, period-determining process in the mammalian circadian clock. *Proc. Natl. Acad. Sci. U.S.A.* 106 15744–15749. 10.1073/pnas.0908733106 19805222 PMC2736905

[B57] Ito H. T. Moser E. I. Moser M. B. (2018). Supramammillary Nucleus modulates spike-time coordination in the prefrontal-thalamo-hippocampal circuit during navigation. *Neuron* 99 576-587.e5. 10.1016/j.neuron.2018.07.021 30092214

[B58] Izhikevich E. M. (2007). *Dynamical Systems in Neuroscience : The Geometry of Excitability and Bursting.* Cambridge, MA: MIT Press.

[B59] Jahnsen H. Llinas R. (1984). Ionic basis for the electro-responsiveness and oscillatory properties of guinea-pig thalamic neurones in vitro. *J. Physiol.* 349 227–247. 10.1113/jphysiol.1984.sp015154 6737293 PMC1199335

[B60] Jouvet M. (1969). Biogenic amines and the states of sleep. *Science* 163 32–41. 10.1126/science.163.3862.32 4303225

[B61] Kim E. J. Jeong D. U. (1999). Transdermal scopolamine alters phasic REM activity in normal young adults. *Sleep* 22 515–520. 10.1093/sleep/22.4.515 10389227

[B62] Kirk I. J. McNaughton N. (1991). Supramammillary cell firing and hippocampal rhythmical slow activity. *Neuroreport* 2 723–725. 1810464 10.1097/00001756-199111000-00023

[B63] Kirk I. J. McNaughton N. (1993). Mapping the differential effects of procaine on frequency and amplitude of reticularly elicited hippocampal rhythmical slow activity. *Hippocampus* 3 517–525. 10.1002/hipo.450030411 8269041

[B64] Kodama T. Takahashi Y. Honda Y. (1990). Enhancement of acetylcholine release during paradoxical sleep in the dorsal tegmental field of the cat brain stem. *Neurosci. Lett.* 114 277–282. 10.1016/0304-3940(90)90576-u 2402335

[B65] Konopacki J. Bland B. H. MacIver M. B. Roth S. H. (1987a). Cholinergic theta rhythm in transected hippocampal slices: independent CA1 and dentate generators. *Brain Res.* 436 217–222. 10.1016/0006-8993(87)91664-7 3435823

[B66] Konopacki J. MacIver M. B. Bland B. H. Roth S. H. (1987b). Carbachol-induced EEG ‘theta’ activity in hippocampal brain slices. *Brain Res.* 405 196–198. 10.1016/0006-8993(87)91009-2 3567594

[B67] Lauriello J. Kenny W. M. Sutton L. Golshan S. Ruiz C. Kelsoe J. (1993). The cholinergic REM sleep induction test with pilocarpine in mildly depressed patients and normal controls. *Biol. Psychiatry* 33 33–39. 10.1016/0006-3223(93)90275-i 8420594

[B68] Le Bon-Jego M. Yuste R. (2007). Persistently active, pacemaker-like neurons in neocortex. *Front. Neurosci.* 1:123–129. 10.3389/neuro.01.1.1.009.2007 18982123 PMC2518052

[B69] Lee M. G. Chrobak J. J. Sik A. Wiley R. G. Buzsaki G. (1994). Hippocampal theta activity following selective lesion of the septal cholinergic system. *Neuroscience* 62 1033–1047. 10.1016/0306-4522(94)90341-7 7845584

[B70] Leonard T. O. Lydic R. (1997). Pontine nitric oxide modulates acetylcholine release, rapid eye movement sleep generation, and respiratory rate. *J. Neurosci.* 17 774–785. 10.1523/jneurosci.17-02-00774.1997 8987799 PMC6573244

[B71] Levey A. I. Edmunds S. M. Koliatsos V. Wiley R. G. Heilman C. J. (1995). Expression of m1-m4 muscarinic acetylcholine receptor proteins in rat hippocampus and regulation by cholinergic innervation. *J. Neurosci.* 15(5 Pt 2), 4077–4092. 10.1523/jneurosci.15-05-04077.1995 7751967 PMC6578239

[B72] Li C. Y. Poo M. M. Dan Y. (2009). Burst spiking of a single cortical neuron modifies global brain state. *Science* 324 643–646. 10.1126/science.1169957 19407203 PMC2913066

[B73] Llinas R. R. (1988). The intrinsic electrophysiological properties of mammalian neurons: insights into central nervous system function. *Science* 242 1654–1664. 10.1126/science.3059497 3059497

[B74] Loomis A. L. Harvey E. N. Hobart G. A. (1937). Cerebral states during sleep, as studied by human brain potentials. *J. Exp. Psychol.* 21 127–144. 10.1037/h0057431

[B75] Lu J. Sherman D. Devor M. Saper C. B. (2006). A putative flip-flop switch for control of REM sleep. *Nature* 441 589–594. 10.1038/nature04767 16688184

[B76] Luppi P. H. Clement O. Fort P. (2013). Paradoxical (REM) sleep genesis by the brainstem is under hypothalamic control. *Curr. Opin. Neurobiol.* 23 786–792. 10.1016/j.conb.2013.02.006 23490549

[B77] Manseau F. Danik M. Williams S. (2005). A functional glutamatergic neurone network in the medial septum and diagonal band area. *J. Physiol.* 566(Pt 3), 865–884. 10.1113/jphysiol.2005.089664 15919710 PMC1464770

[B78] Marshall L. Helgadottir H. Molle M. Born J. (2006). Boosting slow oscillations during sleep potentiates memory. *Nature* 444 610–613. 10.1038/nature05278 17086200

[B79] McCarley R. W. Hobson J. A. (1975). Neuronal excitability modulation over the sleep cycle: a structural and mathematical model. *Science* 189 58–60. 10.1126/science.1135627 1135627

[B80] McCarthy A. Wafford K. Shanks E. Ligocki M. Edgar D. M. Dijk D. J. (2016). REM sleep homeostasis in the absence of REM sleep: effects of antidepressants. *Neuropharmacology* 108 415–425. 10.1016/j.neuropharm.2016.04.047 27150557

[B81] McCormick D. A. Bal T. (1997). Sleep and arousal: thalamocortical mechanisms. *Annu. Rev. Neurosci.* 20 185–215. 10.1146/annurev.neuro.20.1.185 9056712

[B82] McCormick D. A. McGinley M. J. Salkoff D. B. (2015). Brain state dependent activity in the cortex and thalamus. *Curr. Opin. Neurobiol.* 31 133–140. 10.1016/j.conb.2014.10.003 25460069 PMC4375098

[B83] Mesulam M. M. Mufson E. J. Wainer B. H. Levey A. I. (1983). Central cholinergic pathways in the rat: an overview based on an alternative nomenclature (Ch1-Ch6). *Neuroscience* 10 1185–1201. 10.1016/0306-4522(83)90108-2 6320048

[B84] Meuth S. G. Kanyshkova T. Meuth P. Landgraf P. Munsch T. Ludwig A. (2006). Membrane resting potential of thalamocortical relay neurons is shaped by the interaction among TASK3 and HCN2 channels. *J. Neurophysiol.* 96 1517–1529. 10.1152/jn.01212.2005 16760342

[B85] Mikhail C. Vaucher A. Jimenez S. Tafti M. (2017). ERK signaling pathway regulates sleep duration through activity-induced gene expression during wakefulness. *Sci. Signal.* 10:eaai9219. 10.1126/scisignal.aai9219 28119463

[B86] Miyamoto D. Hirai D. Fung C. C. Inutsuka A. Odagawa M. Suzuki T. (2016). Top-down cortical input during NREM sleep consolidates perceptual memory. *Science* 352 1315–1318. 10.1126/science.aaf0902 27229145

[B87] Miyazaki S. Liu C. Y. Hayashi Y. (2017). Sleep in vertebrate and invertebrate animals, and insights into the function and evolution of sleep. *Neurosci. Res.* 118 3–12. 10.1016/j.neures.2017.04.017 28501499

[B88] Neske G. T. (2015). The slow oscillation in cortical and thalamic networks: mechanisms and functions. *Front. Neural. Circuits* 9:88. 10.3389/fncir.2015.00088 26834569 PMC4712264

[B89] Niwa Y. Kanda G. N. Yamada R. G. Shi S. Sunagawa G. A. Ukai-Tadenuma M. (2018). Muscarinic acetylcholine receptors Chrm1 and Chrm3 Are essential for REM sleep. *Cell Rep.* 24 2231.e7–2247.e7. 10.1016/j.celrep.2018.07.082 30157420

[B90] Oddie S. D. Bland B. H. Colom L. V. Vertes R. P. (1994). The midline posterior hypothalamic region comprises a critical part of the ascending brainstem hippocampal synchronizing pathway. *Hippocampus* 4 454–473. 10.1002/hipo.450040408 7874237

[B91] Ode K. L. Katsumata T. Tone D. Ueda H. R. (2017). Fast and slow Ca(2+)-dependent hyperpolarization mechanisms connect membrane potential and sleep homeostasis. *Curr. Opin. Neurobiol.* 44 212–221. 10.1016/j.conb.2017.05.007 28575719

[B92] Penn Y. Segal M. Moses E. (2016). Network synchronization in hippocampal neurons. *Proc Natl. Acad. Sci. U.S.A* 113 3341–3346. 10.1073/pnas.1515105113 26961000 PMC4812773

[B93] Petrovic M. M. Nowacki J. Olivo V. Tsaneva-Atanasova K. Randall A. D. Mellor J. R. (2012). Inhibition of post-synaptic Kv7/KCNQ/M channels facilitates long-term potentiation in the hippocampus. *PLoS One* 7:e30402. 10.1371/journal.pone.0030402 22348007 PMC3278412

[B94] Petsche H. Stumpf C. Gogolak G. (1962). The significance of the rabbit’s septum as a relay station between the midbrain and the hippocampus. I. The control of hippocampus arousal activity by the septum cells. *Electroencephalogr. Clin. Neurophysiol.* 14 202–211. 10.1016/0013-4694(62)90030-5 14038334

[B95] Pignatelli M. Beyeler A. Leinekugel X. (2012). Neural circuits underlying the generation of theta oscillations. *J. Physiol. Paris* 106 81–92. 10.1016/j.jphysparis.2011.09.007 21964249

[B96] Qin K. Dong C. Wu G. Lambert N. A. (2011). Inactive-state preassembly of G(q)-coupled receptors and G(q) heterotrimers. *Nat. Chem. Biol.* 7 740–747. 10.1038/nchembio.642 21873996 PMC3177959

[B97] Rauniar G. P. Gitanjali B. Shashindran C. (1998). Comparative effects of hyoscine butylbromide and atropine sulphate on sleep architecture in healthy human volunteers. *Indian J. Physiol. Pharmacol.* 42 395–400. 9741655

[B98] Renouard L. Billwiller F. Ogawa K. Clement O. Camargo N. Abdelkarim M. (2015). The supramammillary nucleus and the claustrum activate the cortex during REM sleep. *Sci. Adv.* 1:e1400177. 10.1126/sciadv.1400177 26601158 PMC4640625

[B99] Riemann D. Hohagen F. Bahro M. Lis S. Stadmuller G. Gann H. (1994). Cholinergic neurotransmission, REM sleep and depression. *J. Psychosom. Res.* 38(Suppl. 1), 15–25. 10.1016/0022-3999(94)90132-5 7799246

[B100] Sakai K. Crochet S. Onoe H. (2001). Pontine structures and mechanisms involved in the generation of paradoxical (REM) sleep. *Arch. Ital. Biol.* 139 93–107. 11256190

[B101] Sanchez-Vives M. V. McCormick D. A. (2000). Cellular and network mechanisms of rhythmic recurrent activity in neocortex. *Nat. Neurosci.* 3 1027–1034. 10.1038/79848 11017176

[B102] Saper C. B. Fuller P. M. (2017). Wake-sleep circuitry: an overview. *Curr. Opin. Neurobiol.* 44 186–192. 10.1016/j.conb.2017.03.021 28577468 PMC5531075

[B103] Scammell T. E. Arrigoni E. Lipton J. O. (2017). Neural circuitry of wakefulness and sleep. *Neuron* 93 747–765. 10.1016/j.neuron.2017.01.014 28231463 PMC5325713

[B104] Shi Y. F. Han Y. Su Y. T. Yang J. H. Yu Y. Q. (2015). Silencing of cholinergic basal forebrain neurons using archaerhodopsin prolongs slow-wave sleep in mice. *PLoS One* 10:e0130130. 10.1371/journal.pone.0130130 26151909 PMC4495063

[B105] Shi S. Ueda H. R. (2018). Ca(2+) -dependent hyperpolarization pathways in sleep homeostasis and mental disorders. *Bioessays* 40 10.1002/bies.201700105 29205420

[B106] Shinohara Y. Koyama Y. M. Ukai-Tadenuma M. Hirokawa T. Kikuchi M. Yamada R. G. (2017). Temperature-sensitive substrate and product binding underlie temperature-compensated phosphorylation in the clock. *Mol. Cell* 67 783.e20–798.e20. 10.1016/j.molcel.2017.08.009 28886336

[B107] Shu Y. Hasenstaub A. McCormick D. A. (2003). Turning on and off recurrent balanced cortical activity. *Nature* 423 288–293. 10.1038/nature01616 12748642

[B108] Simon A. P. Poindessous-Jazat F. Dutar P. Epelbaum J. Bassant M. H. (2006). Firing properties of anatomically identified neurons in the medial septum of anesthetized and unanesthetized restrained rats. *J. Neurosci.* 26 9038–9046. 10.1523/JNEUROSCI.1401-06.2006 16943562 PMC6675331

[B109] Sirota A. Montgomery S. Fujisawa S. Isomura Y. Zugaro M. Buzsaki G. (2008). Entrainment of neocortical neurons and gamma oscillations by the hippocampal theta rhythm. *Neuron* 60 683–697. 10.1016/j.neuron.2008.09.014 19038224 PMC2640228

[B110] Sitaram N. Wyatt R. J. Dawson S. Gillin J. C. (1976). REM sleep induction by physostigmine infusion during sleep. *Science* 191 1281–1283. 10.1126/science.176724 176724

[B111] Steriade M. Contreras D. Curro Dossi R. Nunez A. (1993a). The slow (1 Hz) oscillation in reticular thalamic and thalamocortical neurons: scenario of sleep rhythm generation in interacting thalamic and neocortical networks. *J. Neurosci.* 13 3284–3299. 10.1523/jneurosci.13-08-03284.1993 8340808 PMC6576531

[B112] Steriade M. McCormick D. A. Sejnowski T. J. (1993b). Thalamocortical oscillations in the sleeping and aroused brain. *Science* 262 679–685. 10.1126/science.8235588 8235588

[B113] Steriade M. Nunez A. Amzica F. (1993c). A novel slow (1 Hz) oscillation of neocortical neurons in vivo: depolarizing and hyperpolarizing components. *J. Neurosci.* 13 3252–3265. 10.1523/jneurosci.13-08-03252.1993 8340806 PMC6576541

[B114] Steriade M. Nunez A. Amzica F. (1993d). Intracellular analysis of relations between the slow (1 Hz) neocortical oscillation and other sleep rhythms of the electroencephalogram. *J. Neurosci.* 13 3266–3283. 10.1523/jneurosci.13-08-03266.1993 8340807 PMC6576520

[B115] Sunagawa G. A. Sumiyama K. Ukai-Tadenuma M. Perrin D. Fujishima H. Ukai H. (2016). Mammalian reverse genetics without crossing reveals Nr3a as a short-sleeper gene. *Cell Rep.* 14 662–677. 10.1016/j.celrep.2015.12.052 26774482

[B116] Suzuki S. Rogawski M. A. (1989). T-type calcium channels mediate the transition between tonic and phasic firing in thalamic neurons. *Proc. Natl. Acad. Sci. U.S.A.* 86 7228–7232. 10.1073/pnas.86.18.7228 2550936 PMC298030

[B117] Tatsuki F. Ode K. L. Ueda H. R. (2017). Ca(2+)-dependent hyperpolarization hypothesis for mammalian sleep. *Neurosci. Res.* 118 48–55. 10.1016/j.neures.2017.03.012 28433628

[B118] Tatsuki F. Sunagawa G. A. Shi S. Susaki E. A. Yukinaga H. Perrin D. (2016). Involvement of Ca(2+)-dependent hyperpolarization in sleep duration in mammals. *Neuron* 90 70–85. 10.1016/j.neuron.2016.02.032 26996081

[B119] Teles-Grilo Ruivo L. M. Baker K. L. Conway M. W. Kinsley P. J. Gilmour G. Phillips K. G. (2017). Coordinated acetylcholine release in prefrontal cortex and hippocampus is associated with arousal and reward on distinct timescales. *Cell Rep.* 18 905–917. 10.1016/j.celrep.2016.12.085 28122241 PMC5289927

[B120] Teles-Grilo Ruivo L. M. Mellor J. R. (2013). Cholinergic modulation of hippocampal network function. *Front. Synaptic. Neurosci.* 5:2. 10.3389/fnsyn.2013.00002 23908628 PMC3726829

[B121] Thinschmidt J. S. Kinney G. G. Kocsis B. (1995). The supramammillary nucleus: is it necessary for the mediation of hippocampal theta rhythm? *Neuroscience* 67 301–312. 10.1016/0306-4522(95)00045-k 7675171

[B122] Tomita J. Nakajima M. Kondo T. Iwasaki H. (2005). No transcription-translation feedback in circadian rhythm of KaiC phosphorylation. *Science* 307 251–254. 10.1126/science.1102540 15550625

[B123] Ukai H. Kiyonari H. Ueda H. R. (2017). Production of knock-in mice in a single generation from embryonic stem cells. *Nat. Protoc.* 12 2513–2530. 10.1038/nprot.2017.110 29189772

[B124] Van Dort C. J. Zachs D. P. Kenny J. D. Zheng S. Goldblum R. R. Gelwan N. A. (2015). Optogenetic activation of cholinergic neurons in the PPT or LDT induces REM sleep. *Proc. Natl. Acad. Sci. U.S.A.* 112 584–589. 10.1073/pnas.1423136112 25548191 PMC4299243

[B125] Venkatakrishnan A. J. Deupi X. Lebon G. Tate C. G. Schertler G. F. Babu M. M. (2013). Molecular signatures of G-protein-coupled receptors. *Nature* 494 185–194. 10.1038/nature11896 23407534

[B126] Vertes R. P. Kocsis B. (1997). Brainstem-diencephalo-septohippocampal systems controlling the theta rhythm of the hippocampus. *Neuroscience* 81 893–926. 9330355 10.1016/s0306-4522(97)00239-x

[B127] Walter W. G. (1938). Critical review: the technique and application of electro-encephalography. *J. Neurol. Psychiatry* 1 359–385. 10.1136/jnnp.1.4.359 21610936 PMC1088109

[B128] Wang Z. Ma J. Miyoshi C. Li Y. Sato M. Ogawa Y. (2018). Quantitative phosphoproteomic analysis of the molecular substrates of sleep need. *Nature* 558 435–439. 10.1038/s41586-018-0218-8 29899451 PMC6350790

[B129] Weber F. Chung S. Beier K. T. Xu M. Luo L. Dan Y. (2015). Control of REM sleep by ventral medulla GABAergic neurons. *Nature* 526 435–438. 10.1038/nature14979 26444238 PMC4852286

[B130] Weber F. Dan Y. (2016). Circuit-based interrogation of sleep control. *Nature* 538 51–59. 10.1038/nature19773 27708309

[B131] Weng F. J. Williams R. H. Hawryluk J. M. Lu J. Scammell T. E. Saper C. B. (2014). Carbachol excites sublaterodorsal nucleus neurons projecting to the spinal cord. *J. Physiol.* 592 1601–1617. 10.1113/jphysiol.2013.261800 24344163 PMC3979614

[B132] Wess J. Liu J. Blin N. Yun J. Lerche C. Kostenis E. (1997). Structural basis of receptor/G protein coupling selectivity studied with muscarinic receptors as model systems. *Life Sci.* 60 1007–1014. 10.1016/s0024-3205(97)00041-6 9121341

[B133] Williams J. H. Kauer J. A. (1997). Properties of carbachol-induced oscillatory activity in rat hippocampus. *J. Neurophysiol.* 78 2631–2640. 10.1152/jn.1997.78.5.2631 9356412

[B134] Woodnorth M. A. Kyd R. J. Logan B. J. Long M. A. McNaughton N. (2003). Multiple hypothalamic sites control the frequency of hippocampal theta rhythm. *Hippocampus* 13 361–374. 10.1002/hipo.10111 12722977

[B135] Xu M. Chung S. Zhang S. Zhong P. Ma C. Chang W. C. (2015). Basal forebrain circuit for sleep-wake control. *Nat. Neurosci.* 18 1641–1647. 10.1038/nn.4143 26457552 PMC5776144

[B136] Yoder R. M. Pang K. C. (2005). Involvement of GABAergic and cholinergic medial septal neurons in hippocampal theta rhythm. *Hippocampus* 15 381–392. 10.1002/hipo.20062 15630696

[B137] Yoshida K. Shi S. Ukai-Tadenuma M. Fujishima H. Ohno R. I. Ueda H. R. (2018). Leak potassium channels regulate sleep duration. *Proc. Natl. Acad. Sci. U.S.A.* 115 E9459–E9468. 10.1073/pnas.1806486115 30224462 PMC6176580

[B138] Zant J. C. Kim T. Prokai L. Szarka S. McNally J. McKenna J. T. (2016). Cholinergic neurons in the basal forebrain promote wakefulness by actions on neighboring non-cholinergic neurons: an opto-dialysis study. *J. Neurosci.* 36 2057–2067. 10.1523/JNEUROSCI.3318-15.2016 26865627 PMC4748083

